# The Potential of Dietary Bioactive Compounds against SARS-CoV-2 and COVID-19-Induced Endothelial Dysfunction

**DOI:** 10.3390/molecules27051623

**Published:** 2022-03-01

**Authors:** Jack N. Losso

**Affiliations:** School of Nutrition and Food Sciences, Louisiana State University, Baton Rouge, LA 70803, USA; jlosso@agcenter.lsu.edu

**Keywords:** endothelium dysfunction, SARS-CoV-2, COVID-19, functional foods, food bioactives

## Abstract

COVID-19 is an endothelial disease. All the major comorbidities that increase the risk for severe SARS-CoV-2 infection and severe COVID-19 including old age, obesity, diabetes, hypertension, respiratory disease, compromised immune system, coronary artery disease or heart failure are associated with dysfunctional endothelium. Genetics and environmental factors (epigenetics) are major risk factors for endothelial dysfunction. Individuals with metabolic syndrome are at increased risk for severe SARS-CoV-2 infection and poor COVID-19 outcomes and higher risk of mortality. Old age is a non-modifiable risk factor. All other risk factors are modifiable. This review also identifies dietary risk factors for endothelial dysfunction. Potential dietary preventions that address endothelial dysfunction and its sequelae may have an important role in preventing SARS-CoV-2 infection severity and are key factors for future research to address. This review presents some dietary bioactives with demonstrated efficacy against dysfunctional endothelial cells. This review also covers dietary bioactives with efficacy against SARS-CoV-2 infection. Dietary bioactive compounds that prevent endothelial dysfunction and its sequelae, especially in the gastrointestinal tract, will result in more effective prevention of SARS-CoV-2 variant infection severity and are key factors for future food research to address.

## 1. SARS-CoV-2 Infection, COVID-19, and Endothelial Dysfunction

COVID-19 is an endothelial disease associated with endothelial dysfunction, which has been recognized as crucial in the pathogenesis and progression of COVID-19 [1,2,3,4]. The vascular endothelium is affected directly and indirectly by SARS-CoV-2 infection. The endothelium is affected directly during infection through the expression and function of its receptor angiotensin-converting enzyme 2 (ACE2) in the vasculature. The endothelium is affected indirectly in the recruitment of inflammatory leukocytes that contribute to tissue damage and cytokine release, both of which are drivers of acute respiratory distress syndrome (ARDS). SARS-CoV-2-and COVID-19-associated dysfunctional endothelium is characterized by dysregulation and disruption of endothelial homeostasis toward a pro-inflammatory phenotype, hyperproduction of inflammatory cytokines including IL-6, IL-8, and TNF-α and dysregulation of coagulation, vascular tone, endothelial permeability, and vascular inflammation.

The SARS-CoV-2 virus that causes COVID-19 can infect several organs including the lungs, brain, intestines, kidneys, heart, and blood vessels [5,6]. Histological analyses of COVID-19-related cases have revealed endothelial injuries in the lungs, heart, and kidney [7]. As a result, a dysfunctional endothelium has a negative impact on the control of hemostasis, fibrinolysis, vasomotion, inflammation, and vascular permeability [1]. Before and during acute COVID-19, clinical conditions including aging, physical inactivity, obesity, diabetes, and hypertension, which are risk factors for severe SARS-CoV-2 infection, are independent risk factors for endothelial dysfunction. These clinical conditions become major risk factors for severe SARS-CoV-2 infection and COVID-19 development and progression. Therefore, risk factors for endothelial dysfunction in non-COVID-19 conditions such as obesity and diabetes become risk factors for or exacerbate COVID-19 infection severity. This knowledge is important for a better understanding of the effects of clinical conditions such as metabolic syndrome on endothelial homeostasis before and during SARS-CoV-2 infection and possible implications for targeted preventions.

The endothelium, a monolayer of cells constituting the inner lining of arteries, veins, and capillaries throughout the circulatory system, weighs approximately 1800 g and represents the largest endocrine organ in the human body. Under normal homeostatic conditions, it forms a semi-permeable barrier that prevents leaking of excessive plasma fluid and regulates selective delivery of nutrients and hormones to underlying tissues [8,9]. Once considered a mere physical barrier between circulating blood components and underlying tissues, the endothelium is now recognized as an important modulator of vascular function. With the growing understanding of endothelial mediators and their role, it has become increasingly clear that endothelial abnormalities may represent an early sign not only of hemodynamic diseases, but also of metabolic disturbances.

The several functions of endothelium may vary, in part, depending on the vessel structure and district location within the body. In conduit vessels such as the aorta, the endothelial cell surface prevents adhesion of platelets and monocytes, reduces the release of pro-inflammatory cytokines, and limits the activation of clotting. In resistance arteries, the endothelium physiologically regulates regional blood flow and modulates systemic blood pressure [10].

In response to both humoral and mechanical stimuli, the vascular endothelium contributes to the regulation of blood flow and blood pressure by release of vasodilators such as nitric oxide (NO), prostacyclin (PGI2) or endothelium-derived hyperpolarizing factor (EDHF), and vasoconstrictors such as endothelin-1 (ET-1), prostaglandins (PGH2) and Ang II [11]. Increased blood flow and shear stress produce endothelium-mediated vasodilation through the release of NO [11]. This gaseous molecule represents the predominant vasodilator released from endothelial cells, and is also involved in inhibition of platelet aggregation, leukocyte adhesion, and vascular smooth muscle cell migration and proliferation [12]. NO is produced via the enzymatic conversion of L-arginine to citrulline by endothelial NO synthase (eNOS), whose protein expression and activity are modulated by multiple stimuli. Endothelial dysfunction is a condition characterized by reduced vasodilation resulting from decreased NO bioavailability and increased oxidative stress, and it is usually associated with the pathogenesis of atherosclerosis, hypertension, cardiovascular diseases, and now COVID-19 [4,13,14]. Endothelial dysfunction has been dubbed as the cornerstone in COVID-19 severity [15].

## 2. Risk Factors for Endothelial Cell Dysfunction

Endothelial dysfunction is synonymous with altered endothelial cell phenotype in which the reduced production and bioavailability of NO promote a pro-thrombotic and pro-inflammatory state [16]. During endothelial dysfunction, the decline in NO bioavailability disrupts the physiological homeostasis of the vessel wall and favors the increased, unbalanced bioactivity of vasoconstrictor and pro-atherogenic factors such as ET-1. This predisposes the vessels to leukocyte adhesion, platelet activation, oxidative stress, thrombosis, coagulation, and inflammation, hence promoting the formation and progression of atherogenic plaques [17,18]. Endothelial dysfunction is not only a hallmark of hypertension, atherosclerosis, and coronary heart disease, but also an early feature of aging, and a distinctive aspect of metabolic disorders including insulin resistance, hyperglycemia, and dyslipidemia. Several factors can affect the endothelium including and not limited to aging, oxidative stress, low-density lipoprotein (LDL) oxidation, smoking, hypercholesterolemia, hypertension, chronic hyperglycemia, genetic factors, and the recent SARS-CoV-2 infection that causes COVID-19 pandemic. Dietary factors can also induce endothelial dysfunction. Each factor and its specific effects on endothelial function are briefly described below.

### 2.1. Aging

Aging is one of the main risk factors for dysfunction of both endothelial and vascular smooth muscle cells. Endothelial cell injury is normally mitigated by endogenous reparative processes mediated by bone marrow-derived endothelial progenitor cells (EPCs) [19]. Overtime, the endothelium ability to regenerate itself tends to decrease, partly as a consequence of reduced EPC availability and/or mobilization. Even in the absence of specific risk factors, senescence is accompanied by several structural and functional changes occurring throughout the entire vascular system and contributing to alter endothelial barrier integrity. Moreover, the aging process may damage the balance between vasodilator and vasoconstrictor substances produced by endothelium [20]. Endothelium-dependent vasodilation progressively declines with age and this is due to diminished eNOS expression and NO production in endothelial cells while increased amounts of reactive oxygen species (ROS) and reactive nitrogen species are observed [21,22]. With aging, endothelial dysfunction increases the permeability of endothelium to lipoproteins, monocytes, and macrophages, therefore enhancing smooth muscle cell migration and proliferation and aiding the formation of an intermediate lesion and progression to atherosclerotic plaques [23,24]. With aging, endothelial dysfunction aids in the formation of an intermediate lesion and progression to an atherosclerotic plaque [25,26,27]. The presence of endothelial abnormalities in old people becomes a risk factor not only for the pathogenesis of cardiovascular disease, including atherosclerosis and hypertension [28,29], endothelial dysfunction is also a risk factor for aging-related diseases such as erectile dysfunction, renal failure, circadian cycle alterations, osteoporosis, retinopathy, and Alzheimer’s disease [30,31,32,33,34,35].

### 2.2. Oxidative Stress

Reactive oxygen species (ROS), including hydrogen peroxide (H_2_O_2_) and superoxide anion (O_2_^−^), are produced in a limited amount in endothelial cells under normal physiological conditions. The balance between oxidant and antioxidant processes regulates the amount of ROS generated by endothelial cells, and an imbalance due to an increase in ROS is implicated in vascular dysfunction. Superoxide anion (O_2_^−^) is a powerful and long-lived oxidant which reacts with NO to produce peroxynitrite (ONOO-) and contributes to endothelial barrier dysfunction by promoting vascular hyperpermeability and leukocyte adhesion [8,36]. As a direct consequence, small and dense molecules such as low-density lipoproteins can accumulate in the arterial intima, where the concomitant increased adhesiveness of leukocytes facilitates their entry in endothelial cells and triggers the inflammatory process [37,38].

Oxidative stress may be generated by several pathological conditions such as hyperglycemia, diabetes, hypertension, dyslipidemia, smoking, or high levels of oxidized low-density lipoproteins; all these factors are able to produce ROS, which in turn rapidly inactivate NO [39,40]. The increased ROS generation by mitochondria-mediated and membrane-associated NADPH oxidase activity of xanthine oxidase and myeloperoxidase, the decreased ability of free radical scavengers such as superoxide dismutase (SOD), and the higher susceptibility of macromolecules to free radical damage are among mechanisms involved in endothelial oxidative stress [41,42,43].

### 2.3. Oxidized Low-Density Lipoprotein

Oxidized low-density lipoprotein (Ox-LDL) can induce endothelial dysfunction by several mechanisms including (1) binding to scavenger receptors such as receptor-A1, -A2, and lectin-like oxidized low-density lipoprotein receptor (SR-A1, SR-A2, and LOX-1) [44,45]; (2) upregulating the expression of its own receptor LOX-1 on endothelial cells and activating endothelial cells; (3) promoting the growth and migration of smooth muscle cells, monocytes/macrophages and fibroblasts; and (4) leading to oxidative stress through generation of excessive ROS amounts.

### 2.4. Smoking

Exposure to cigarette smoke increases the rate of cardiovascular diseases by inducing morphological alterations and functional exchanges in endothelial and smooth muscle cells [46]. Smoking causes a progressive downregulation of the endothelial NO synthase (eNOS) enzyme, predisposing the vessels to adhesion of platelets and leukocytes, and promoting thrombus formation [47,48]. A decrease in GSH and 3-nitrotyrosine levels and reduced expression of both Nrf2/ARE and heme oxygenase-1 (HO-1) pathways, as well as glutamate-cysteine ligase catalytic (GCLC), have been demonstrated in endothelial dysfunction of young smokers [49,50]. Chronic smoking favors elevated plasma levels of free fatty acids, vasopressin and serum cholesterol, and concomitantly reduces high-density lipoproteins that may indirectly damage endothelial cells and blood vessels by increasing the permeability to lipids and blood components [51]. Interestingly, increased blood pressure levels and endothelial dysfunction have also been documented in smokeless tobacco consumers [52].

### 2.5. Hypercholesterolemia

Hypercholesterolemia (plasma cholesterol levels > 200 mg/dL) induces endothelial dysfunction in arteries through increased production of O_2_^−^ and near-complete abrogation of vascular NO bioavailability, predisposing to atherosclerosis [53]. Hypercholesterolemia induces endothelial dysfunction in arteries through increased production of O_2_^−^ and near-complete abrogation of vascular NO bioavailability, predisposing to atherosclerosis [54]. Hypercholesterolemia and more specifically high oxidized cholesterol derivatives such as 7-ketocholesterol, 7α-hydroxycholesterol, and 7β-hydroxycholesterol constitute potent inhibitors of endothelium-dependent arterial relaxation and have been considered as links between hypercholesterolemia and endothelial dysfunction [55]. These oxysterols have been shown to be associated with a marked reduction in vasorelaxation, which occurs early before the formation of atherosclerotic lesions [56]. Further, activated monocytes releasing inflammatory mediators (IL-1 and TNF-α) stimulate endothelial cells as well as smooth muscle cells to secrete growth factors that will enhance atherogenesis [57]. Hypercholesterolemia is also associated with the production of asymmetric dimethylarginine (ADMA), an endogenous inhibitor of NOS whose level is inversely related to NO production [58,59].

### 2.6. Hypertension

There is a reciprocal relationship between abnormal endothelial function and hypertension. Studies have shown that in patients with essential hypertension, the decreased NO bioavailability in endothelial cells results from increased oxidative stress and subsequent activation of signaling pathways related to inflammation and contraction in vascular smooth muscle cells [60,61,62]. ROS may directly alter vascular function and cause changes in vascular tone by enhancing the synthesis and activity of ROS-producing enzymes including the NADPH oxidase and xanthine oxidase, by altering the mitochondrial respiratory chain, and by uncoupling the activity of endothelial NOS [63]. Growing evidence indicates that ROS generated by NADPH oxidase and activation of redox-dependent signaling cascades are produced by angiotensin II (Ang II) signaling in vascular cells [63]. Ang II, acting through the AT1 receptors, stimulates NADPH oxidase, causing the accumulation of superoxide (O_2_^−^), hydrogen peroxide (H_2_O_2_) and peroxynitrite (ONOO^−^) [43,63]. Therefore, abnormal stimulation of AT1 receptor by increased circulating or tissue levels of Ang II can induce endothelial dysfunction and hypertension, as well as inflammatory response. Inhibition of vascular NADPH oxidase induction and subsequent preservation of arterial NO availability during Ang II administration can prevent endothelial dysfunction [64]. Analogously, treatment with angiotensin-converting enzyme inhibitors (ACE-I) or angiotensin receptor blockers to reduce pressure levels in hypertensive patients significantly improves endothelial dysfunction, therefore confirming that hypertension is a risk factor for endothelial dysfunction [65].

## 3. Dietary Risk Factors for Endothelial Dysfunction

### 3.1. Advanced Glycation End Products (AGEs)

**Figure 1:**
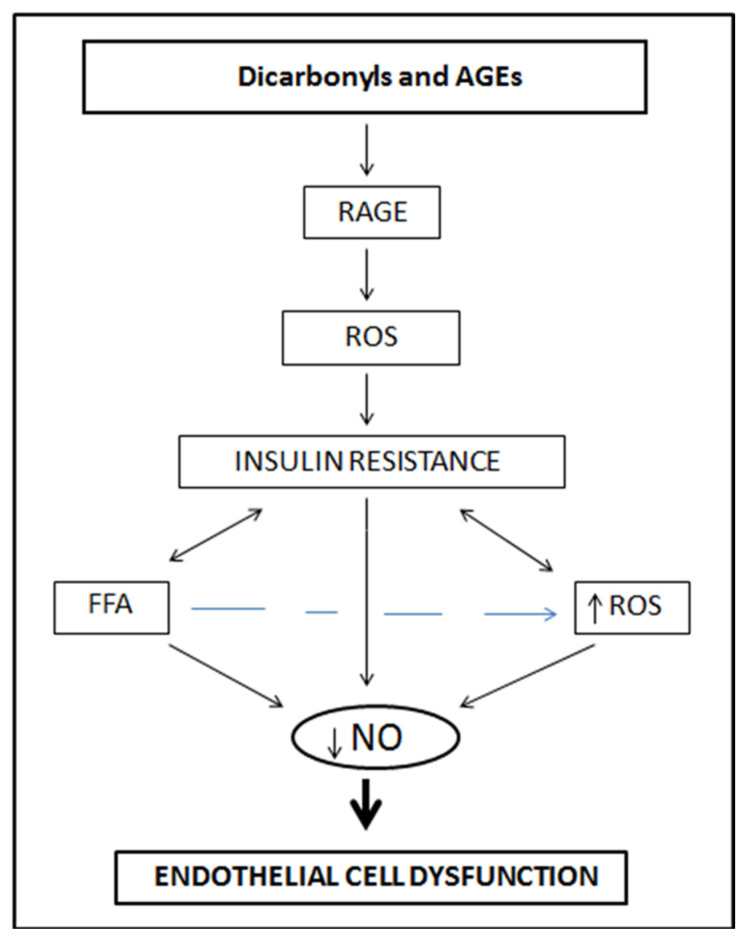
The relationship between dietary risk factor such as advanced glycation end products and endothelial cell dysfunction.

Endothelial dysfunction represents an early, pivotal, and common denominator of vascular and metabolic diseases including obesity and diabetes, both of which are major risk factors for severe COVID-19 infection (Figure 1). A single oral AGE-rich beverage challenge (approximately 1.8 × 10^6^ AGE units) in 44 diabetic and 10 non-diabetic subjects was associated with significant increases in serum AGEs with altered clinical measures of endothelial function in diabetic and non-diabetic subjects [66]. Thus, chronic exposure to high AGE-rich diets can lead to and/or accelerate endothelial dysfunction and vascular disease over time [66]. In patients with type 2 diabetes (T2DM), a high-AGE meal induces a more pronounced acute impairment of vascular function than does an otherwise identical low-AGE meal.

Hyperglycemia is the initiating event in the formation of AGEs, a heterogeneous group of modified proteins, lipids, and nucleic acids formed primarily through non-enzymatic Maillard reactions between amino groups and glucose derivative dicarbonyls [67,68]. Accumulation of AGEs alters the functional property of matrix components and mediates sustained cellular changes. AGEs decrease NO bioavailability and eNOS expression by accelerating eNOS mRNA degradation [69,70]. The receptors of AGEs (RAGE) have been found in many cells including monocytes, macrophages, and endothelial cells, where they mediate cellular migration and upregulation of pro-inflammatory and prothrombotic molecules. Binding of AGEs to their RAGE receptor increases intracellular enzymatic superoxide production [71,72] and promotes macrophage-mediated inflammation in the vessel wall [73].

Soluble RAGE (sRAGE) is an endogenous decoy of RAGE that is inversely associated with RAGE level. AGER1 is the best characterized RAGE so far. The expression of RAGE is upregulated in aorta, retina, and kidney under diabetic conditions, thus contributing to the progression of vasculopathy, retinopathy and vascular nephropathy in diabetic patients. The interaction of circulating AGEs with endothelial RAGE causes increased permeability of endothelial cells and subsequent alteration of endothelial physical integrity [9,74,75]. The covalent binding of AGEs to RAGE is associated with reduced levels of sRAGE, leading to depletion of cellular antioxidant defense mechanisms such as glutathione and vitamin C [76], activation of NADPH oxidase and resulting increased generation of intracellular ROS. These free radicals are able to activate the redox-sensitive nuclear transcription factor NF-κB in vascular wall cells via the phosphorylation of the RAS/ERK pathway. Translocation of NF-κB to the nucleus promotes the expression of NF-kB-regulated genes, culminating with upregulation of inflammatory cytokines such as TNF-α, IL-6 and IL-8; adhesion molecules such as E-selectin, the intercellular adhesion molecule-1 (ICAM-1), and the vascular adhesion molecule (VCAM-1); inflammatory enzymes such as cyclooxygenase-2 (COX-2), and matrix metalloproteinases (MMPs) [9,77,78]. In addition, AGE–RAGE interaction decreases vasculature elasticity quenching NO and inducing the expression of the potent vasoconstrictor ET-1 [79], which contributes to defective endothelium-dependent vasodilation in diabetes [80].

### 3.2. Chronic Hyperglycemia, Reactive Dicarbonyls and Glucotoxicity

Each increase in glucose concentration in the blood is associated with perturbation of endothelial function. Chronic hyperglycemia provides a source of sugar molecules that may promote reducing reactions and be converted by autooxidation into reactive dicarbonyls. Reactive dicarbonyls, such as methylglyoxal (MG), can aggravate endothelial dysfunction by impairing Ser-1177 phosphorylation on the eNOS catalytic site, thereby inhibiting enzyme activity, and decreasing NO production [81], as well as by inducing oxidative stress and production of inflammatory cytokines such as IL-6, IL-1β and TNF-α. Even in healthy subjects, acute ingestion of glucose is associated with a transitory increase in superoxide generation, with increased activity of transcription factors such as nuclear NF-κB and activating protein-1 (AP-1) [82]. In diabetic individuals, chronic hyperglycemia-induced peroxynitrite formation has been shown to generate nitrotyrosines, harmful molecules able to interact and interfere with several distinct signal transduction pathways [83].

### 3.3. Hypertriglyceridemia, Oxidized Ldl, Trans Fatty Acids, Free Fatty Acids and Lipotoxicity

High levels of lipids, including triglycerides (TGs), trans fatty acids, and low-density lipoprotein cholesterol (LDL), damage vascular tissues and their functions. These lipid-induced disturbances are referred to as lipotoxicity [84]. Lipotoxicity, characterized by pathological changes at the cellular and organ levels that result from excess lipids in the circulation or in tissues, is caused in large part by overnutrition. As for glucotoxicity, lipotoxicity may impair endothelial function by a number of related mechanisms, including increased production of ROS, pro-inflammatory signaling, mitochondrial dysfunction which uncouples oxidative phosphorylation in endothelium, endoplasmic reticulum stress, and apoptosis [85]. Regular consumption of high-fat meals raises the peak of circulating lipids and insulin and can impair endothelial function by activating monocytes, and enhancing ROS generation with the downstream signaling cascade leading to activation of the NF-κB inflammatory signaling pathway [86].

Oxidized low-density lipoprotein can induce endothelial dysfunction by binding to lectin-like oxidized low-density lipoprotein receptor-1 (LOX-1): this interaction increases the expression of angiotensin-converting enzyme and reduces the intracellular concentration of NO with mechanisms already described for Ang II [87,88]. Consumption of a single high-fat meal rich in saturated fatty acids can induce endothelial dysfunction in otherwise healthy subjects, as evidenced by the related increased concentrations of VCAM-1, ICAM-1, IL-6, IL-18, and TNF-α [89,90]. As additional evidence of lipotoxicity, it has been shown that incubation of endothelial cells with palmitate, the major saturated fatty acid present in most foods, increases NADPH oxidase expression and stimulates superoxide production [91,92]. A more prolonged or higher intake of food rich in trans fatty acid, as well as sustained high plasma levels of trans fatty acids, may lower HDL cholesterol, and concomitantly increase plasma levels of triglycerides and LDL cholesterol, impair glucose uptake and adversely affect endothelial function mostly by enhancing oxidative stress [83,93]. The assumption that a high intake of trans fatty acids may exacerbate endothelial dysfunction is supported by results obtained from the Nurses’ Health Study I, a cross-sectional study on 730 women, showing that dietary fatty acids may increase concentrations of E-selectin, soluble intercellular adhesion molecule (sICAM-1), and soluble vascular adhesion molecule (sVCAM-1) [94].

### 3.4. Homocysteine and Endothelial Function

Smoking, alcohol consumption, and physical inactivity can elevate homocysteine (Hcy) levels. Hyperhomocysteinemia (H-Hcy) is found more often in the elderly and in men, which may in part be caused by lower serum levels of folic acid and vitamin B12, reduced methionine metabolism, and higher serum creatinine levels in men compared to women [95]. Hyperhomocysteinemia is directly related to vascular endothelial cell damage, which derives from endothelial dysfunction induced by enhanced oxidative stress [95,96]. Oxidative radicals generated by hyperhomocysteinemia inevitably initiate the oxidative degradation of lipids in endothelial cell membrane, leading to loss of membrane function [97]. Similarly, hyperhomocysteinemia-induced superoxide production by NADPH oxidase activity indirectly decreases NO bioavailability by rapid consumption of NO and generation of peroxynitrites [95]. Moreover, hyperhomocysteinemia may reduce NO production by disrupting NO signaling via a mechanism that involves the PI3K/Akt and PKC pathways.

Finally, in vitro studies have shown that elevated concentrations of homocysteine can induce apoptosis of endothelial cells by activating the Fas cell-death pathway, the p53/NOx pathway, and the cytochrome-c-activated caspase 3 and 9 pathways [98].

### 3.5. Obesity

The microvasculature from visceral fat of obese individuals is an important source of low-grade inflammation and oxidative stress. Small vessels or perivascular adipose tissue (PVAT) of obese individuals generate excessive proinflammatory cytokines, including TNF-α. The latter stimulates the production of ROS mainly through NAD(P)H oxidase activation, which leads to reduced NO bioavailability. Adipose tissue inflammation, reduced NO bioavailability, insulin resistance and oxLDL are major causes of endothelial cell injury and dysfunction including imbalance between pro-inflammatory/pro-coagulant and anti-inflammatory/anticoagulant in obesity. The obesity condition is associated with worsening of the microvascular endothelial function as shown by flow-mediated dilation in the brachial arteries of obese individuals [99]. Obese individuals are highly susceptible to severe SARS-CoV-2 infection and severe COVID-19 development through several mechanisms including chronic inflammation, endothelium imbalance, dysregulated immune response, metabolic dysfunction, and dysfunctional mesenchymal stem cells/adipose-derived mesenchymal stem cell [100]. The vascular endothelium of obese individuals may overexpress ACE2, transmembrane protease serine 2 (TMPRSS2), and furin, which make the endothelial cells more vulnerable to severe SARS-CoV-2 infection and impair endothelium balance [100]. Furin is a serine protease that may also be important to support viral entry into cells, subsequent replication inside the cells and the exit of virus particles from cells [101]. All the comorbidities of COVID-19 including hypertension, diabetes, cardiovascular and respiratory disease are tightly associated with obesity [100].

## 4. Identifying Dietary Inhibitors of Endothelial Dysfunction

Since endothelial dysfunction precedes and predicts the development of a wide range of pathological conditions, strategies to prevent or delay the endothelial cell abnormalities may help to protect against the progression of risk factors associated with the severity of SARS-CoV-2 infection and COVID-19. The rationale of preventing endothelial dysfunction with dietary components, rather than focusing on various therapies with antioxidants or diet compounds administered when endothelial dysfunction is already evident, is that several interventional studies aimed at restoring endothelial function with dietary bioactives often show limited improvements and not total recovery [102]. A large number of studies have investigated the role of dietary compounds identified among flavonoids, flavones, terpenes, quinones, anthocyanins, phenolics and fatty acids, and found an inverse association between consumption of these compounds and the presence and degree of endothelial dysfunction.

### 4.1. Flavonoids, Isoflavones, Flavonols, Anthocyanins and Endothelial Function

#### 4.1.1. Flavonoids, Flavonoid Metabolites, and Endothelial Function

Epidemiological and observational studies support a strong linear association between flavonoid consumption and healthy endothelial tissues. Several publications investigate the association of flavonoids and endothelial function in healthy subjects or patients with risk factors for severe COVID-19 infection including obesity, diabetes, or other pathological conditions.

Tea, cocoa, grapes, citrus, onions, wines, berries, pulses, and marine products are very rich sources of flavonoids, and regular consumption of these bioactive dietary compounds under appropriate conditions should protect the endothelium against injury, mainly by decreasing oxidative stress. In fact, in vitro studies have provided evidence that flavonoids protect the endothelium by scavenging superoxide anions, singlet oxygen, and low-density lipoproteins [103,104]. Pycnogenol, a mixture of monomeric (catechin and epicatechin) and polymeric procyanidins as well as phenolic acids, improves endothelial function because it is anti-inflammatory, reduces platelet aggregation, improves microcirculation, and protects against capillary leaking [105]. In addition, flavonoids may increase NO bioavailability by decreasing superoxide-mediated NO breakdown, and several of them may directly enhance NO synthesis and release by upregulating the expression of NO synthase [106,107]. Epidemiological studies have observed that regular moderate consumption of red wine, rich in flavonoids, is associated with increased NO production, or that consumption of cacao and green tea improve endothelial function as a consequence of their flavonoid components, such as catechins [108,109]. A clinical investigation in the inpatient Clinical Research Center of the Brigham and Women’s Hospital enrolled 19 healthy older adults (mean age 72 years) who consumed one packet of Cocoapro^TM^, Mars Inc. containing 451 mg flavonols (84 mg epicatechin, 28 mg of catechin, 339 mg flavonol oligomers, 19 mg caffeine, 204 mg theobromine, and 119 calories) in one cup of water [110]. Flow-mediated vasodilation was measured as reactive hyperemia peripheral arterial tonometry (RH-PAT) taken at 2, 4, 6 and 8 h after Cocoapro^TM^ consumption. Flavonols and their metabolites were measured by HPLC. The results of the study showed a positive correlation between PAT response and flavonoid concentration [110]. Individuals with habitual dietary intake of flavonoids (2000–4500 mg of flavonols per week) had superior endothelial function compared to lower flavonoid consumers. The study also confirmed once again that endothelial function varies within populations, and the variability is in part associated with personal habitual dietary intake of flavonoids.

The health benefits of dark cocoa (commonly referred to as Noir Amertume extreme, or very dark chocolate) appear to be unique despite its content of 10–15 g of saturated fat per serving size. Noir cocoa is bitter but healthier than most of the milk-loaded versions of chocolate on the market. In part, this may be because milk proteins bind to procyanidins and suppress their activities. In addition, most Noir cocoa products appear to have trace amounts of proteins unlike other milk-loaded cocoa products. The beneficial effects of Noir or dark chocolate have been shown in patients with cirrhosis. In a phase 2, double-blind, randomized controlled clinical trial, a significant decrease in hepatic venous pressure gradient (HVPG) and concomitant amelioration of systemic hypotension and endothelial function was observed in 11 patients 30 min after the ingestion of dark chocolate containing 85% cocoa; conversely, patients with cirrhosis receiving white chocolate showed a significant increase in HVPG [111]. The trust in cocoa products is so high that usual consumers would undoubtedly achieve benefits if regulatory agencies could suggest to manufacturers to voluntary remove from the market cocoa products that do not improve endothelial function. Unfortunately, the market is saturated with both high-quality and below-standard cocoa products, making cocoa product evaluation very difficult [112,113].

Black tea flavonoids activate eNOS in endothelial cells, increase NO production and increase cGMP levels. Studies investigating the relationship between green tea consumption and endothelial function concluded that populations consuming green tea on a regular basis (1 or 2 cups a day) had a decreased risk of cardiovascular events [107]. Endothelial dysfunction was found substantially improved in 20 healthy smokers who consumed 400 mL of green tea containing 24.72 mg of epigallocatechin gallate (EGCG) and 59.24 mg of other catechins [114]. In chronic young smokers (*n* = 20), consumption of 8 g per day of green tea improved the number of circulating endothelial progenitor cells (EPCs) as determined by flow cytometry [115]. The health benefits of green tea were associated with its content of EGCG, which enhances endothelial NO production by multiple mechanisms. Other health benefits associated with black or green tea flavonoid consumption relate to the improvement in NO-dependent brachial artery flow-mediated dilation. Reverse of vascular endothelial dysfunction by green tea catechins in hypertension has been ascribed to catechins ability to inhibit NF-κB activation, VCAM and ICAM activation, leukocyte adhesion to endothelial cells and leukocyte penetration into the intima by MCP-1, IL-8, and E-selectin.

Adzuki beans and peanut skins are good sources of procyanidins [115,116,117]. Although the interactions of adzuki bean procyanidins and endothelial cells have never been investigated, it is straightforward to suggest that procyanidins from adzuki may be helpful to improve endothelial functions when used appropriately.

#### 4.1.2. Isoflavones and Endothelial Function

Soybean is a major source of isoflavones including genistein and daidzein. Isoflavones can be found in several soy products including tofu, soymilk, natto, and soymilk whey, depending on the matrix. Studies demonstrating the health benefits of soy isoflavones on endothelial function are numerous [118]. In one study, endothelial function measured by flow-mediated vasodilation (FMD) of brachial artery showed a significant improvement in postmenopausal women with metabolic syndrome (n = 20) treated with 54 mg/day of genistein for 6 months along with a Mediterranean-style diet (25–30% fat, <10% saturated fatty acids, 55–60% carbohydrates and 15% protein) [119]. Postmenopausal women (n = 15) consuming Konako soy (toasted ground soy containing 12.95 mg of soy protein + 50 mg isoflavone) showed a significant increase in NO accompanied by a decrease in blood pressure levels [120].

#### 4.1.3. Flavonols and Endothelial Function

Quercetin has vasorelaxant and antioxidative properties. In human endothelial cells, quercetin or its metabolite isorhamnetin inhibit the expression of biomarkers of endothelial dysfunction including VCAM-1, ICAM-1 and MCP-1 at a physiologically attainable concentration of 2 µM [121]. In healthy men (n = 23) consuming 4.3 g of onion extract containing 51 mg of quercetin for 30 days, endothelial function was significantly improved, as indicated by the postprandial FMD value increasing from 5.1 ± 2.2% to 6.7 ± 2.6% [122]. Conversely, in a double-blind cross-over study evaluating the effects of 8 week administration of quercetin (150 mg) or placebo in 49 males with an APOE genotype, quercetin was effective at increasing HDL cholesterol and reducing both waist circumference and systolic blood pressure but had no effect on endothelial function [123].

#### 4.1.4. Anthocyanins and Endothelial Function

Several in vitro and animal studies have demonstrated the potential of anthocyanins to modulate endothelial function. Studies in humans are rare. The metabolites/catabolites in berries, such as simple phenolic acids, are probably the in vivo bioactive compounds that may have effects on the endothelium [124].

### 4.2. Stilbenes and Endothelial Function

*Trans*-resveratrol (3,5,4′-trihydroxy-trans-stilbene) is the most investigated stilbene. It can be found in grapes, red wines, peanut skins and other food sources. Resveratrol improves endothelial function and enhances NO production by several mechanisms, including stimulation of eNOS phosphorylation, eNOS-mRNA and BH4, and decreasing ADMA and the acylated and less active form of eNOSeNOS-Ac [125]. Pre-clinical studies have confirmed that *trans*-resveratrol can inhibit endothelial dysfunction by enhancing the bioavailability of resveratrol [126]. In a double-blind randomized cross-over study, 19 overweight/obese men or postmenopausal women were assigned to groups consuming 30, 90 or 270 mg of resveratrol. Plasma resveratrol levels reached 181, 532 and 1232 ng/mL, respectively. At these nanomolar concentrations, resveratrol increased eNOS and FMD from 4.1% to 6.6% and 6.6% to 7.7%, respectively. One important factor that may limit resveratrol efficacy is its solubility. This stilbene is easily soluble in pure ethanol, suggesting that wines with 14% alcohol do not carry significant amounts of resveratrol. Nevertheless, studies using resveratrol supplementations have in most cases shown that resveratrol provides a prolonged improvement in FMD following repeated administration [126].

### 4.3. Terpenes, Terpene Saponins and Endothelial Function

Saponins protect endothelial cells from dysfunction. Most of the data on saponins and endothelial cell function have been obtained using ginseng or licorice. Ginseng saponins Rb1 and Rg1 from panax ginseng improve endothelial cell function by modulating the activity of PI3K/Akt/eNOs and l-arginine transport in endothelial cells [127]. Ginsenoside Rb1 reverses homocysteine-induced endothelial cell dysfunction through eNOS downregulation [128]. Glycyrrhizic acid is a triterpenoid saponin glycoside found in Licorice and used to prevent diabetic vascular complications. Glycyrrhizic acid inhibits AGEs-induced endothelial dysfunction [129]. Saponins protect endothelial cells from oxidized LDL-induced cell injury. Other sources of dietary saponins including asparagus and fenugreek have never been investigated for their potential protective effects on the endothelium.

### 4.4. Quinones and Endothelial Function

Anthraquinones including emodin, aloe-emodin, rhein, chrysophanol and physcion are bioactive compounds present in rhubarb and Cassia *alata* [130,131]. Emodin, aloe-emodin and rhein are pleiotropic molecules that interact with and inhibit several biomarkers of endothelial function [130]. Emodin was reported to have potential to treat COVID-19 through its ability to act on a wide range of disease targets including BCL2L1, PTGS2, TP53, CASP3, CXCL8, EGFR, CSF2, MAPK14, FNT, VEGFA, MCL1, MAPK1, and 1L1B [132].

Thymoquinone is the major bioactive molecule in black seed (*Nigella sativa*). Thymoquinone can protect constitutive and induced NOS from degradation by pyrogallol, resulting in increased NO production and improved endothelial function [133].

Coenzyme Q10 (CoQ10) has important protective effects in endothelium. As mentioned above, Ang II is a major risk factor associated with the pathogenesis of hypertension and cardiovascular disease. In vitro, Ang II is able to increase ROS, increase the expression of p22 (phox) and Nox2 subunits of NADPH oxidase, upregulate ICAM-1 and VCAM-1, inhibit insulin-induced NO production, and overall impair endothelial function [134]. Coenzyme Q10 at 1–10 µM dose dependently inhibits Ang II activities in vitro. In vivo, CoQ10 reduced superoxide production and recouples mitochondrial oxidative phosphorylation [135]. In patients with type 2 diabetes, CoQ10 quenches ROS, reduces superoxide production and improves endothelial function by increasing brachial artery FMD [136]. CoQ_10_ plays an important role in cellular ATP production but is decreased in cardiovascular disease and influenza infected patients. The possibility of C0Q10 alleviating inflammation and myocardial inflammation in COVID-19 has been raised [137].

### 4.5. Olive Oil and Endothelial Function

Olive oil is to the Mediterranean diet what soybean is to the Japanese diet. It is omnipresent in meals and consumed in high amounts. The oil contains several bioactive compounds including oleic acid, oleanolic acid, oleuropein, oleocanthal, taxifolin, hydroxytyrosol, homovanillyl alcohol, caffeic and ferulic acid. Several in vivo studies involving small and large numbers of participants have investigated the health benefits of olive oil, and several of these studies have confirmed the beneficial effects of consuming olive oil [138]. In a study at Mayo Clinic, designed to investigate the long-term effect of olive oil and/or green tea supplementation on endothelial function, participants (n = 82 started and n = 52 completed the study) with early atherosclerosis and presence of endothelial dysfunction (EndoPAT score < 2.0) were randomized to receive 30 mL of polyphenol-rich olive oil alone or in association with green tea EGCG for 4 months [139]. To determine the effect of olive oil or olive oil and EGCG on endothelial function, Endo-PAT and inflammatory biomarkers including hsCRP, IL-6, sICAM-1 and sVCAM-1, and oxidative stress biomarkers including oxLDL and 8-isoprostane, were measured in the 52 patients who completed this study. The results showed that the consumption of olive oil alone significantly improved endothelial function in patients with low to intermediate risk of atherosclerosis, with no additional beneficial effects associated with EGCG. Several olive oil constituents have potent anti-inflammatory activities and can restrict the progression of various inflammation-linked diseases ranging from endothelial dysfunction to arthritis, cancer, and the severity of COVID-19 [140,141].

### 4.6. Curcuminoids and Endothelial Function

Age is the major risk factor for declining endothelial function. Menopausal women are not exempt from age-associated endothelial function decline. Thirty-two menopausal women were enrolled in a study that evaluated the effect of curcumin vs. exercise on endothelial function [142]. The participants were divided into 3 groups: a control group, a group that underwent moderate aerobic training for 8 weeks and a group of participants that ingested curcumin for 8 weeks. Flow-mediated dilation was measured as a marker of endothelial function and showed that curcumin was equally as effective as exercise in improving endothelial function in postmenopausal women. In a recent pilot study, the effect of curcumin phytosome (Meriva) was evaluated on the progression of diabetic microangiopathy [143]. Twenty-five diabetic patients received and ingested two tablets containing 1 g Meriva per tablet daily for four weeks [143]. Meriva is a water-soluble curcumin-phosphatidylcholine conjugate that has better solubility properties for human studies and uses than curcumin.

Endothelial dysfunction is an early marker of atherosclerosis. Flow-mediated dilation (FMD), measured by ultrasonography, is used to non-invasively assess endothelial dysfunction. Preparations of curcumin may improve FMD and thus endothelial dysfunction [144].

### 4.7. Factors That Reduce Hyperhomocysteinemia

Methionine converts to homocysteine in absence of folate and B12 vitamin, suggesting that a combination of both reducing the consumption of methionine-rich foods and taking adequate amounts of folate and B12 vitamin should help reduce the risk of developing hyperhomocysteinemia. Foods rich in melatonin such as pistachios, tart cherries, almonds, and sunflower seeds, and foods rich in betaine such as quinoa or spinach, can help prevent the buildup of homocysteine.

## 5. Dietary Inhibitors of Endothelial Dysfunction and SARS-CoV-2 Infection

Increasing evidence demonstrates that endothelial dysfunction is a risk factor and predictor of SARS-CoV-2 infection and COVID-19 severity. The correlation between the severity of SARS-CoV-2 infection and COVID-19 progression has been demonstrated in individuals with dysfunctional endothelium including obese, diabetic, hypertensive, and immunocompromised people. Preventing endothelial dysfunction may delay or weaken SARS-CoV-2 infection.

Section 4 of this review provided dietary bioactives that maintain endothelial homeostasis; the current section lists dietary bioactives that have shown in vitro inhibitory activities of SARS-CoV-2 infection and have potential for in vivo inhibition of the virus and attenuation of the COVID-19 disease. Edible marine polysaccharides including fucoidan, carrageenan, and chondroitin sulfate have been investigated. Plant terpenoids such as glycyrrhizic acid are also being studied.

Fucoidan is a heterogeneous group of sulfated polysaccharide with a high content of l-fucose. Brown algae and marine invertebrates such as sea cucumber are major sources of fucoidans. Fucoidan is a good candidate for prevention and inhibition of endothelial dysfunction in metabolic syndrome and SARS-CoV-2 infection [145]. Fucoidan antioxidant activity, complement inhibition and interaction with the vascular endothelial growth factor (VEGF) are of high interest for application in SARS-CoV-2 infection and COVID-19 progression. Interest in using fucoidan in protecting endothelial cell homeostasis has so far received little attention. Fucoidan induces NO production by activating and enhancing eNOS and AkT phosphorylation, scavenges ROS, induces Nrf2, binds to VEGF_165_ and reduces VEGF_165_ expression and VEGF_165_ receptor expression and activation [146,147]. Fucoidan also binds C1q, C4 and inhibits CFB/C3 binding [146]. Fucoidan can reduce the vascular inflammation and oxidative stress caused by iNOS expression [147].

Carrageenan (Iota, λ, and κ-carrageenan) a polymer from marine alga *Rhodophyceae* is variably used as an emulsifier, a stabilizer, colloid, thickener, suspender, syneresis control, or gelling that improves the consistency of several foods and beverages including infant formula, dairy products, soymilk, chocolate and flavored milk, and nutritional supplement beverages. Carrageenan exhibits high antioxidant, free radical scavenging, antibacterial, anticoagulant, and immunomodulatory activity [148]. Several in vitro studies indicate that λ-carrageenan inhibits several enveloped RNA viruses including SARS-CoV-2 with an EC_50_ value of 0.9 ±  1.1 μg/mL by preventing viral attachment to cell surface receptors and virus entry [149]. Iota-carrageenan combined with ivermectin and nasally administered improved the outcome in COVID-19 patients in clinical trial NCT04425850 completed in Argentina [150]. Clinical trials NCT04590365 (UK), NCT04681001 (Austria), and NCT04793984 (Austria) for iota-carrageenan alone have recruited and are in progress [150]. Other studies have shown that Iota and λ-carrageenan had potent antiviral activity (EC_50_ between 3.2 and 7.5 µg/mL, respectively) and carrageenan and griffithsin combination exhibited synergistic activity with very low EC_50_ values between 0.2 and 3.8 µg/mL; and a combination index < 1 against recent SARS-CoV-2 mutations [151]. Carrageenan specifically interacts with the viral envelop of glycoprotein gp120 and inhibits the interaction of the virus with CD4 [150].

Glycyrrhizic acid (GLR) is a terpene saponin food additive with FDA Generally Recognized as Safe (GRAS) status. GLR has anti-inflammatory, antioxidative, antiallergenic, antimicrobial, antiviral including HIV, antiparasite, and anticancer properties. GLR has the ability to prevent SARS-CoV-2 viral infection of endothelial cells by binding to the ACE2 receptor [152,153].

## 6. Current and Future Directions

Endothelial dysfunction occurs in the early stages of several chronic degenerative diseases including COVID-19. Glucotoxicity, lipotoxicity or glucolipotoxicity caused by chronic consumption of high levels of reducing sugars or high levels of saturated fats impairs endothelial cells in various tissues and sets the tone for chronic disease including SARS-CoV-2 infection and severe COVID-19 development and progression. Metabolic syndrome is a major risk factor associated with endothelial dysfunction with or without SARS-CoV-2 infection. However, SARS-CoV-2 infection of subjects with severe defective endothelial cells leads to severe COVID-19 progression. The correlation between metabolic syndrome and the severity of SARS-CoV-2 infection and COVID-19 development has been established.

Since the gut is also a site of infection and the ACE2 receptor for SARS-CoV-2 is found in the gastrointestinal tract, dietary treatment has a role in reducing SARS-CoV-2 infection through the gastrointestinal tract. Although SARS-CoV-2 is mostly a respiratory disease, the lung–gut axis has been identified and exacerbates/accelerates COVID-19 progression. Dietary bioactives can protect endothelial cells in the gastrointestinal tract and bioavailable dietary bioactives can protect endothelial cells in other organs.

In clinical trial NCT04521322, 394 healthy hospital personnel including physicians, nurses, kinesiologists, and other healthcare providers dedicated to care of COVID-19 patients were randomly assigned to receive a nasal spray containing iota-carrageenan (196 participants) or placebo (saline solution, 198 participants) for 21 days at 10 hospitals in Argentina [154]. COVID-19 developed in 1.0% (2 out of 196) of participants who received the iota-carrageenan spray and COVID-19 developed in 5% (10 out of 198) of participants who received the placebo. The primary end point was clinical COVID-19, as confirmed by RT-PCR. Iota-carrageenan was safe and effective in preventing COVID-19 disease in hospital workers who care for COVID-19 patients.

Preventing and reversing endothelial dysfunction appear to be feasible, as shown by some of the work cited in Section 4 and Section 5. Prevention of endothelial dysfunction may weaken SARS-CoV-2 infection and subsequent COVID-19 disease. Well-designed healthy diets can help prevent endothelium dysfunction. Current and future efforts in food research and product development should consider vascular dysfunction as an important target in healthy eating because improving vascular function helps prevent endothelial dysfunction, metabolic syndrome, and reduces the severity of SARS-CoV-2 infection. Potential dietary bioactives that address vascular system dysfunction and its sequelae in comorbidities such as obesity may have an important role in reducing SARS-CoV-2 infection and its long-lasting effects. Identifying biomarkers of endothelial dysfunction in clinics should help medical practitioners to work with food scientists and designers in developing foods that help prevent viral infectivity and progression. Other viruses worse than SARS-CoV-2 and COVID-19 may be awaiting in nature.

## Data Availability

Not applicable.
